# Length-Change Patterns of the Collateral Ligaments During Functional Activities After Total Knee Arthroplasty

**DOI:** 10.1007/s10439-020-02459-3

**Published:** 2020-01-23

**Authors:** S. H. Hosseini Nasab, C. R. Smith, P. Schütz, P. Damm, A. Trepczynski, R. List, W. R. Taylor

**Affiliations:** 1 Institute for Biomechanics, ETH Zürich, Leopold-Ruzicka-Weg 4, 8093 Zurich, Switzerland; 2 Julius Wolff Institute, Charité – Universitätsmedizin Berlin, Berlin, Germany; 3 Human Performance Lab, Schulthess Clinic, Zurich, Switzerland

**Keywords:** TKA, Fluoroscopy, Multibody model, MCL, LCL, Elongation

## Abstract

This study aimed to quantify the elongation patterns of the collateral ligaments following TKA during functional activities of daily living. Using mobile video-fluoroscopy to capture radiographic images of the knee in a group of six patients, each with an ultra-congruent knee implant, tibiofemoral kinematics were reconstructed throughout complete cycles of level gait, downhill walking, stair descent, and squat activities. Kinematic data were then used to drive subject-specific multibody knee models to estimate length-change patterns of the LCL as well as three bundles of the MCL. In addition, a sensitivity analysis examined the role of the attachment site in the elongation patterns. Our data indicate a slackening of the LCL but non-uniform length-change patterns across the MCL bundles (ranging from lengthening of the anterior fibers to shortening of the posterior fibers) with increasing knee flexion angle. Near-isometric behavior of the intermediate fibers was observed throughout the entire cycle of the studied activities. These length-change patterns were found to be largely consistent across different activities. Importantly, length-change patterns were critically sensitive to the location of the femoral attachment points relative to the femoral component. Thus, in TKA with ultra-congruent implants, implantation of the femoral component may critically govern post-operative ligament function.

## Introduction

The importance of proper tensioning of the collateral ligaments for the clinical success of total knee arthroplasty (TKA) has been comprehensively established.^1^,^5^,^35^ A number of different methods for intraoperative ligament balancing have been introduced, targeting tension balance in the medial and lateral collateral ligaments (MCL and LCL) at full extension as well as at 90° of knee flexion.^12^,^33^,^35^ However, the optimal ligament tension (or indeed laxity), during TKA is generally judged based on subjective manual evaluation by the surgeon rather than quantitative metrics.^32^,^33^ Postoperative joint stiffness, accelerated implant wear, and pain have all been associated with over tensioning of the ligaments,^3^,^8^,^31^ whereas excessively lax ligaments contribute to joint instability,^47^ which is frequently reported as a cause of TKA failure.^1^,^12^

To define the optimal ligament tension during TKA and avoid postoperative complications, a thorough understanding of the length-change patterns experienced by the collateral ligaments in natural and replaced knees during different functional activities is crucial. However, such knowledge is extremely limited due to a lack of studies assessing their *in vivo* and dynamic functionality. The few studies that have assessed *in vivo* length-change patterns of the MCL and LCL have generally exploited image-based approaches, combining static video-fluoroscopy and 3D modelling of the knee joint in order to track the relative movement of the ligament attachment sites, and thereby estimate their length-change patterns.^29^,^37^,^38^ Here, Park and co-workers measured healthy subjects performing a forward lunge activity and reported slight lengthening of the MCL anterior bundle and shortening of the MCL posterior bundle and LCL with increasing knee flexion.^38^ Using a similar approach, Liu and co-workers assessed the stance phase of treadmill walking in healthy subjects and found a positive relationship between the length of the MCL anterior bundle and the knee flexion angle, whereas a shortening of the posterior bundle was observed.^29^ The only study that investigated length-change patterns of the collateral ligaments in TKA subjects was performed by Park and co-workers.^37^ Here, the post-TKA MCL and LCL length-change patterns during single-legged lunge were very similar to those reported for healthy knees.^38^ However, all of these studies have used stationary fluoroscopy which clearly limits the imaging capture volume, and thus the range of activities that can be investigated. Recently, our lab has developed a moving fluoroscopy system that now allows the knee joint to be tracked, thus providing access to the assessment of accurate tibio-femoral kinematics throughout complete dynamic activities of daily living.^17^,^28^

In addition to accurate knowledge of subject-specific kinematics, image-based estimation of ligament elongation patterns requires the ligament attachment sites to be accurately identified. Previous *ex vivo* studies have comprehensively described the morphology of the collateral ligament attachment sites,^25^,^26^,^34^,^42^ however such information for living subjects is only accessible through medical imaging of the knee, using e.g. magnetic resonance imaging (MRI). Importantly, uncertainty in identification of the ligament attachment sites using such imaging modalities may substantially affect the reliability of the estimations of ligament elongation patterns.^9^,^41^ However, a clear understanding of the elongation patterns of the MCL and LCL throughout complete functional activities of daily living that considers possible errors induced by uncertainty in ligament attachment sites, remains critically lacking.

This study aimed to assess the length-change patterns of the collateral ligaments of TKA patients throughout level walking, downhill walking, stair descent, and body-weight squat based on the comprehensive kinematic and anatomical measurements gained within the CAMS-Knee datasets.^48^

## Methods

For this analytical, observational cohort study (level of evidence III), six subjects (5 m, 1f, aged 68 ± 5 years, mass 88 ± 12 kg, height 173 ± 4 cm) each with an ultra-congruent knee implant (INNEX FIXUC, Zimmer, Switzerland) were measured 64 to 87 months post-operatively within the CAMS-Knee project.^48^ The study was approved by ethics committees of ETH Zürich (EK 2013-N-90) and Charité-Universitätsmedizin Berlin (EA4/069/06) and all subjects provided written informed consent prior to participation.

All subjects had size M tibial components. Apart from one subject with a size M, all other subjects had a size M^+^ femoral component. A mechanically aligned TKA was targeted for all subjects, however postoperative assessment indicated 3.8° ± 1.8° varus limb alignment. When needed, minimal release of the anterior fibres of the MCL was performed to ensure intraoperative tension balance between the collateral ligaments.

### Subject-Specific Knee Models

**Figure 1:**
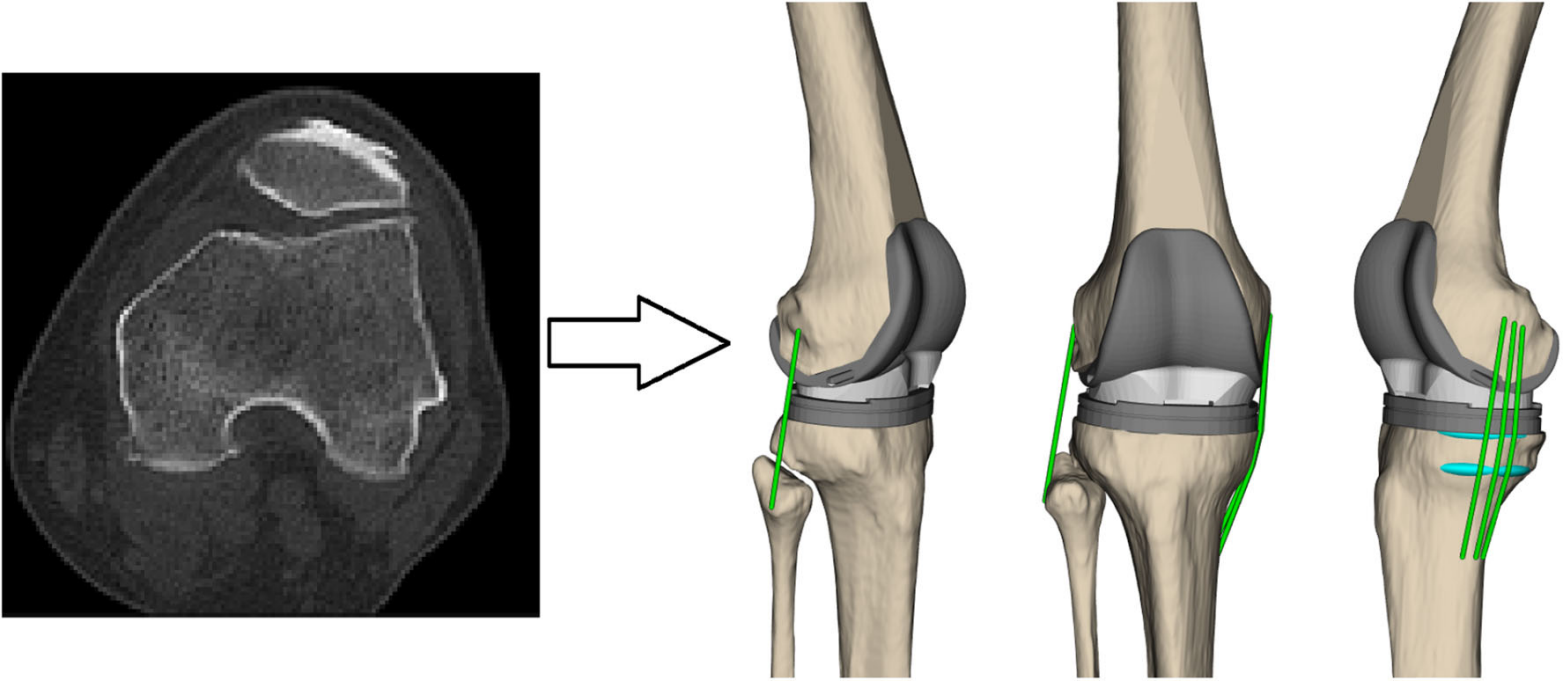
An exemplary pre-operative CT slice (left) together with a subject-specific multi-body model of the knee (right), including the collateral ligament bundles (shown in green) as well as the wrapping surfaces used to prevent penetration of the ligament bundles into the bones (shown in cyan).

The implanted knee of each subject was scanned pre- and post-operatively using a computed tomography (CT) scanner (General Electric - Light Speed 64, 0.6 mm slice intervals in the transverse plane; Fig. 1). The preoperative images were imported into a 3D visualization and modelling software (Amira, Visage Imaging, Berlin, Germany), and 3D geometrical models of the knees were reconstructed. Implant components were superimposed on the knee models, regarding their position and orientation relative to the bones, which were obtained from postoperative CT images.

Based on previous anatomical studies,^25^,^26^,^34^,^42^ the centroids of the femoral and tibial attachment sites of the LCL and intermediate MCL (iMCL) bundle were identified on the subject-specific bone geometries. The bony attachment sites of the anterior MCL (aMCL) and the posterior MCL (pMCL) bundles relative to the iMCL attachments were then determined based on the reported average width of the ligament attachment sites (11.8 mm for the femoral and 14.9 mm for the tibial attachment).^30^

Using the 3D bone geometries together with the implantation data, subject-specific multi-body models were developed in OpenSim.^13^ Here, the OpenSim environment was used only as a platform for geometrical analysis of the ligament fibres, while no musculoskeletal simulation of the loading conditions was performed. The ligament bundles were represented by one-dimensional strands connecting their origin and insertion points (Fig. 1). Due to its small cross sectional area and attachment area,^26^,^34^ a single strand was used to characterize the LCL, while three strands were used to represent the aMCL, iMCL, and pMCL bundles of the MCL.

To avoid penetration of the ligaments into the bone structures and thereby mimic the real curvilinear path of the ligament bundles, virtual wrapping surfaces were defined on the distal femur as well as the proximal tibial bone.

### Video-Fluoroscopy

**Figure 2:**
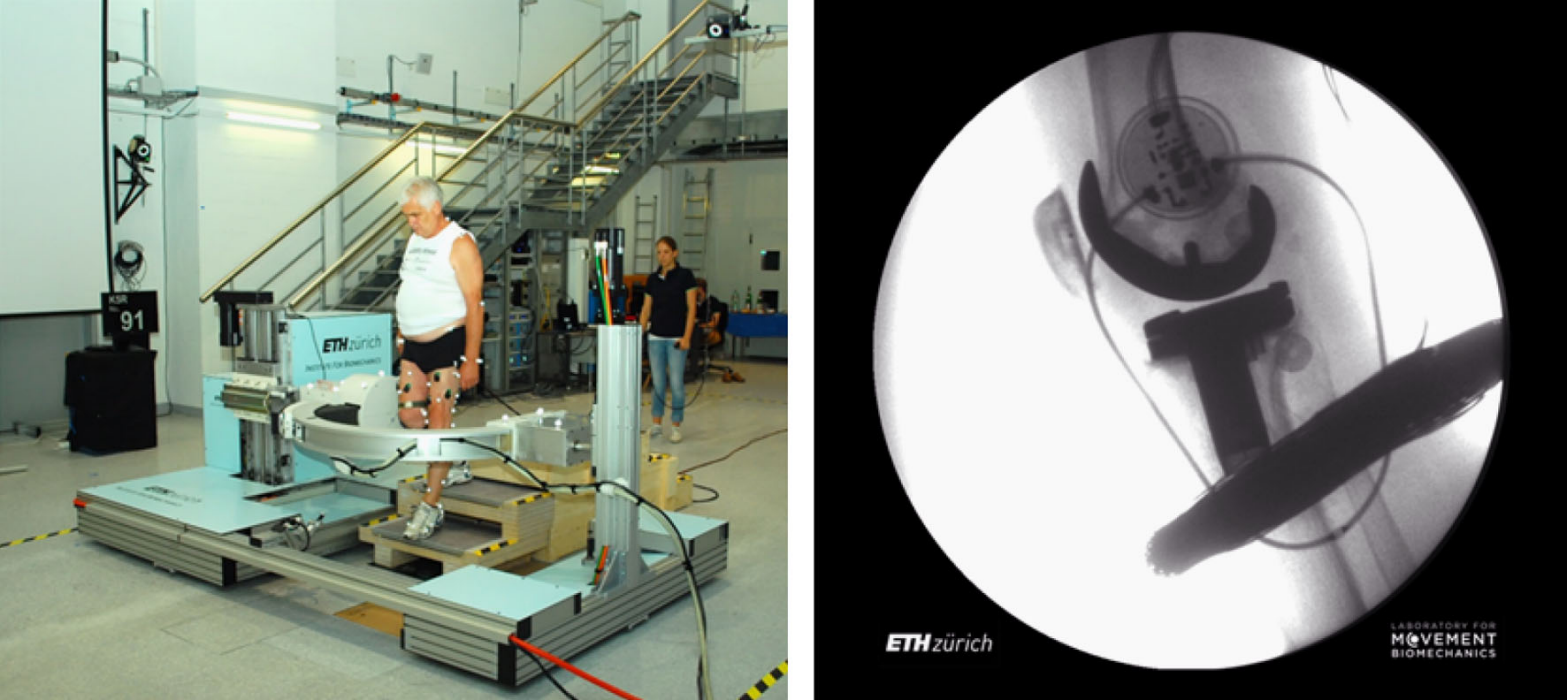
The ETH moving fluoroscope was used to capture radiographic images of the knee joints throughout a range of dynamic activities (left). An exemplary fluoroscopic image including the implant component projections is shown on the right. Specific informed consents were acquired from the subjects in order to publish their images.

The video-fluoroscopy data was captured as a part of the CAMS-Knee project, where each subject performed multiple repetitions of different activities.^48^ The ETH moving fluoroscope^17^,^28^ was used to capture radiographic images of the knee joint throughout entire cycles of level walking, downhill walking, stair descent and body-weight squat (Fig. 2). Three dimensional CAD models of the implant components were then registered to the 2D fluoroscopic images to obtain the accurate tibio-femoral kinematics (registration errors < 1° for rotations and < 1 mm for in-plane translations^17^).

### Ligament Elongation Patterns

The reconstructed tibio-femoral kinematics were used to drive the subject-specific multi-body models, such that the length of ligament bundles could be analyzed throughout the complete activity cycles. Here, length of the ligament bundle was calculated as the length of the curvilinear pathway starting from the ligament origin, passing over the wrapping surfaces (representing bone surfaces), and ending at the ligament insertion. The ligament length-change patterns were calculated as a percentage of the reference length and presented against the corresponding normalized time or implant flexion angle. The reference length of each ligament bundle was defined as the length at heel strike averaged across all trials of level walking.

### Statistical Analysis

A one-way repeated-measures ANOVA, based on statistical parametric mapping^39^ was performed to compare the length-change patterns of the ligament bundles across different activities. Significance was set at *α* =0.05.

### Sensitivity Analysis

A Monte Carlo analysis was performed to assess the influence of the ligament attachment sites on the ligament elongation patterns. The ligament attachment points for a single subject were randomly perturbed from their nominal location based on Gaussian distributions (standard deviations: 5 mm in the anteroposterior and proximodistal directions and 2 mm in the mediolateral (ML) direction) to generate 500 new models. The baseline tibiofemoral kinematics from the squat activity were fed into the random models and the resultant length-change patterns were analyzed. The Pearson correlation coefficient (*r*) between the maximum length-change of the ligament bundles and the shift in the position of their attachment points, as well as, the slope of linear best-fit were calculated.

## Results

### Ligament Elongation Patterns

**Figure 3:**
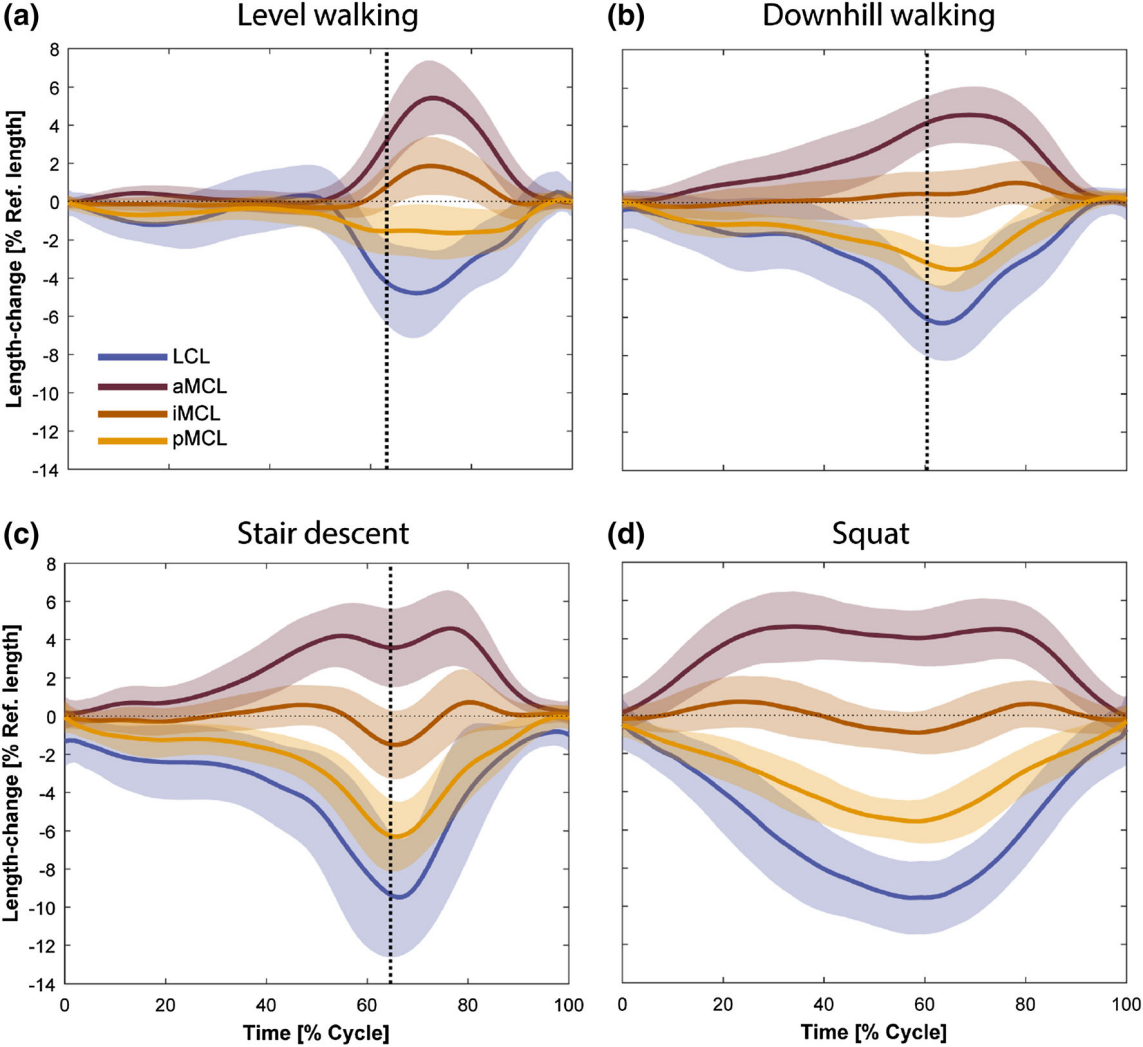
Average elongation patterns of the LCL and three MCL bundles (solid lines represent inter-subject means and shadings represent ± 1 inter-subject standard deviation). The vertical dotted line represents the average toe-off time for the six subjects.

**Figure A1:**
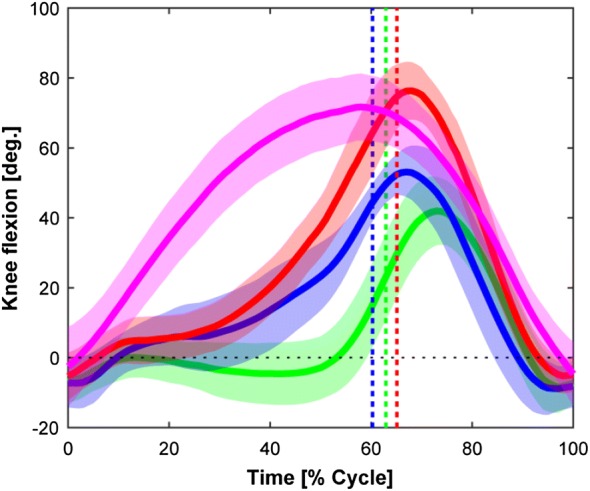
Average implant flexion angles obtained from video-fluoroscopy (solid lines represent inter-subject means and shading represents ± 1 inter-subject standard deviation) during level walking (green), downhill walking (blue), stair-descent (red) and body-weight squat (magenta). The vertical dotted line represents the average toe-off time for the six subjects. Implant flexion is expressed in the joint coordinate system introduced by Grood and Suntay^18^

The average reference lengths of the ligament bundles measured at heel strike of level gait were as follows: 89.2 ± 4.2 mm for aMCL, 88.6 ± 4.3 mm for iMCL, 88.2 ± 4.6 mm for pMCL, and 59.9 ± 4.1 mm for LCL. Collateral ligaments remained close to isometric during the first 50% of the level gait cycle (Fig. 3). From push-off to mid-swing, the LCL showed a rapid slackening (− 4.8 ± 2.5%) and reached its shortest length at around 70% of the gait cycle (GC), where the knee exhibited its largest flexion angle (42.0 ± 9.7°, Fig. A1). During the same period, the aMCL experienced considerable lengthening (5.4 ± 2.1%), the iMCL was only slightly elongated (1.9 ± 1.56%), and the pMCL exhibited a minor shortening (− 1.6 ± 1.5%). Knee extension through terminal swing phase resulted in continuous elongation of the LCL and pMCL, together with shortening of the iMCL and aMCL. As a result, all ligament bundles recovered their reference lengths before the following heel strike.

Length-change patterns of the collateral ligaments during downhill walking and stair decent indicated no significant elongation or shortening of the iMCL (max. length-change: 1.0 ± 1.1% and − 1.5 ± 1.7% for downhill walking and stair decent). However, the other bundles demonstrated bi-phasic length-change patterns including continuous slackening of the LCL (up to − 6.3 ± 1.9% for downhill walking and − 9.5 ± 3.0% for stair descent) and pMCL (up to − 3.5 ± 1.2% for downhill walking and − 6.3 ± 1.9% for stair descent) until 70% GC, with steady lengthening of these bundles thereafter. Conversely, the aMCL was stretched to 4.6 ± 1.5% during downhill walking and similarly 4.6 ± 2.0% during stair descent, with recovery to its reference length by the time of the next heel strike.

During squatting, the LCL slackened with increasing the knee flexion until it reached its shortest length (− 9.6 ± 2.0%) at deep knee flexion (71.7 ± 9.6°, Fig. A1 in Appendix). The MCL bundles exhibited considerably different length-change patterns depending on the fiber location. The aMCL had a rapid lengthening in the first 35% of the squat cycle (with a max. lengthening of 4.6 ± 1.9%) followed by an isometric phase during the next 40% but finally recovered to its reference length during the last 20% of the cycle. The iMCL had almost no length-change throughout the entire squat cycle (with a maximum length-change of − 0.9 ± 1.2%). In contrast, the pMCL exhibited clear shortening with increasing knee flexion angle, reaching its shortest length (− 5.6 ± 1.1%) at deep flexion.

**Figure 4:**
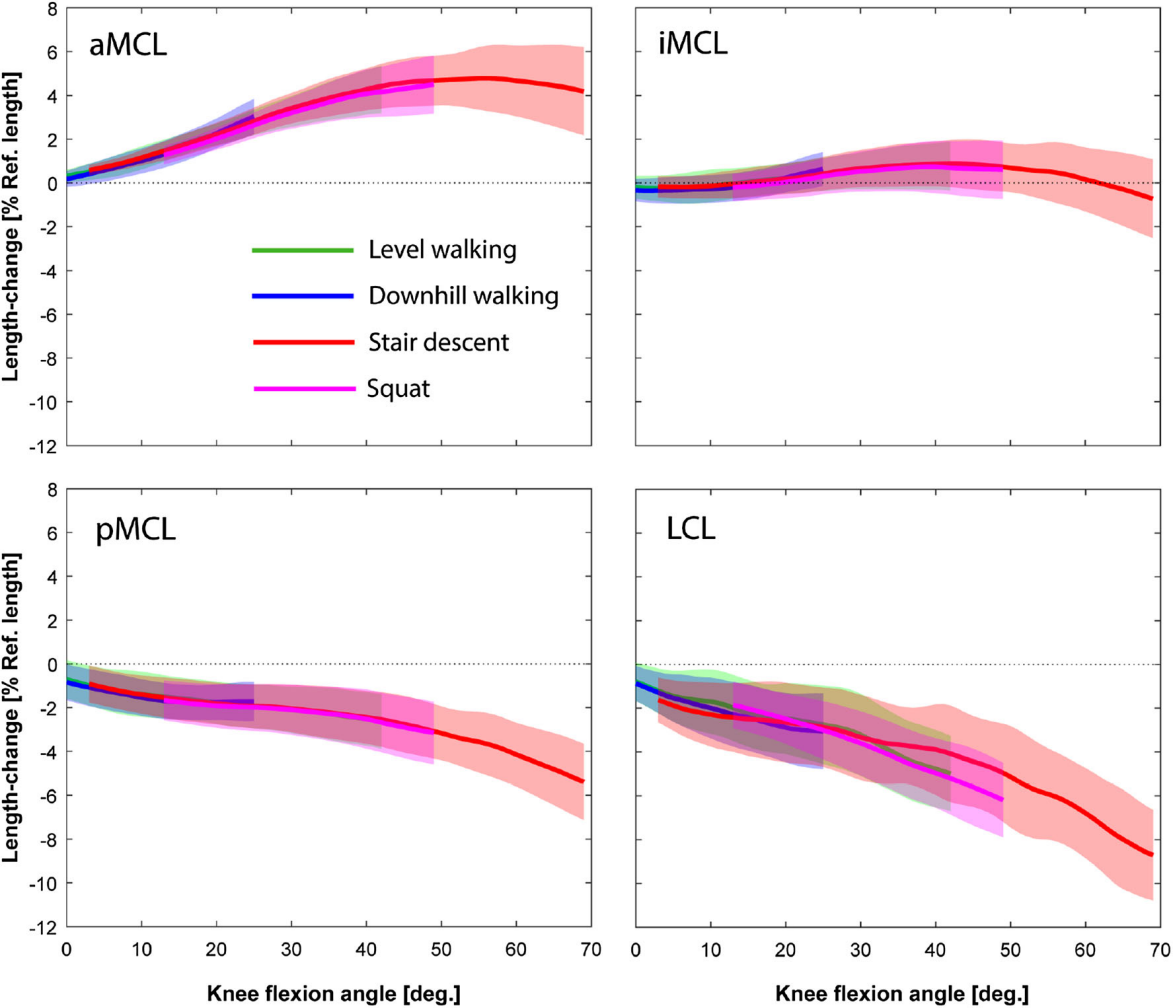
Average length-change patterns of the LCL and MCL bundles during five repetitions of the level walking, downhill walking, stair descent, and squat plotted against knee flexion angle. Average patterns were calculated only over the flexion ranges achieved by all the subjects during all the trials. Solid lines represent inter-subject means while shading displays ± 1 inter-subject standard deviation.

In general, the length-change pattern of the aMCL indicated a positive relationship with the knee flexion angle, while the LCL and pMCL slackened with increasing the knee flexion (Fig. 4). The iMCL was generally isometric throughout the range of knee flexion covered by the studied activities. The repeated measures ANOVA based on SPM revealed no significant task-dependency of the ligament length-change patterns (Table A1 in Appendix).

### Sensitivity Analysis

**Figure 5:**
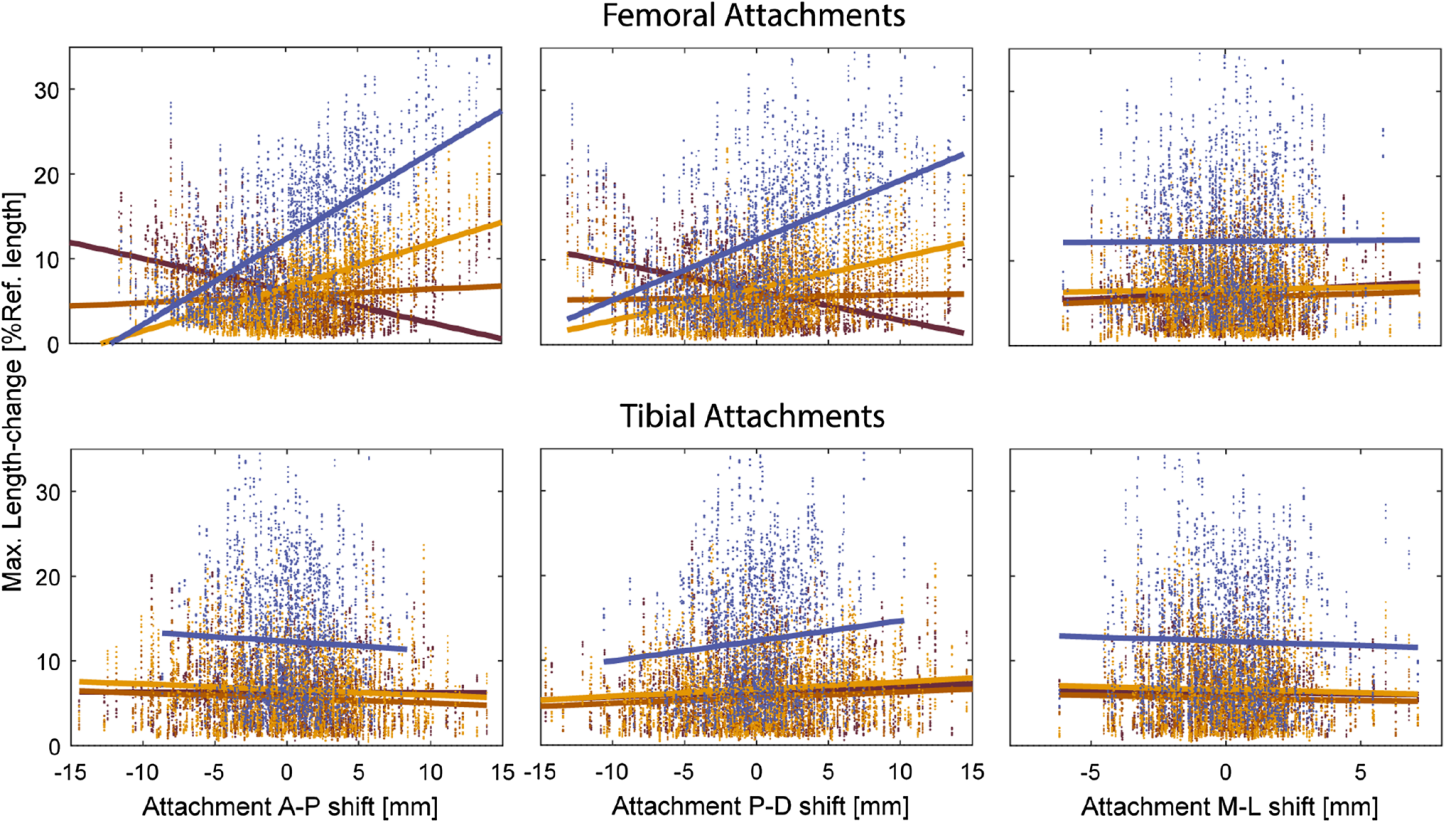
Variation of the maximum length-change of the collateral ligaments during squat for a single subject as a result of moving the femoral (top row) and tibial (bottom row) attachment points in the anteroposterior (left), proximodistal (middle) and mediolateral (right) directions. Solid lines represent the fitted regression lines for LCL (blue), aMCL (dark brown), iMCL (light brown), and pMCL (amber).

The uncertainty analyses on the location of ligament attachment sites revealed that the maximum length-change of the ligaments was most strongly correlated with the anteroposterior (A-P) location of the femoral attachments (Fig. 5). The LCL showed the greatest sensitivity with a 1 mm shift in the A-P direction inducing a 1.1% change in the maximum length-change (*r* = 0.68). The maximum elongation of the aMCL was changed by 0.4% when its femoral attachment was moved anteroposteriorly by 1 mm (*r* = − 0.48). The second largest impact on the maximum length-change of the ligament bundles was due to a variation of the femoral attachments in the proximodistal (P-D) direction (0.7% per mm for the LCL, *r* = 0.48). Perturbing the femoral attachment of the ligament bundles in the mediolateral (M-L) direction had no considerable effect on the length-change patterns. Similarly, the tibial attachment locations did not substantially influence the collateral ligament elongations (sensitivities ranging from − 0.1 to 0.2% per mm (− 0.08 < *r* < 0.10), Fig. 5).

## Discussion

This study reported length-change patterns of the collateral ligaments in a group of patients with an ultra-congruent knee implant design.^48^ Accurate tibio-femoral kinematics were reconstructed from imaging data captured using a moving fluoroscope throughout complete cycles of level gait, downhill walking, stair descent, and squat, and were used to drive subject-specific multibody knee models for the estimation of length-change patterns of the MCL and LCL. Regardless of the activity, our results revealed a slackening of the LCL as well as non-uniform length-change patterns across the MCL bundles (ranging from lengthening of aMCL to shortening of pMCL and isometric behavior of iMCL) with increasing knee flexion angle. These findings provide an indication of the functional behavior of these key ligaments throughout complete cycles of activities of daily living in TKA patients, and thus a basis for understanding the consequences of selective surgical release of ligament fibers. Critically, the ligament length-change patterns were found to be almost exclusively sensitive to the location of the femoral attachment points relative to the femoral component, while the location of the tibial attachment sites were clearly of less importance. These data therefore highlight the key role of femoral (over tibial) component implantation in governing post-operative ligament function.

Until now, our current knowledge on collateral ligament function has been almost exclusively obtained from cadaveric studies. Here, *in vitro* investigations on natural and replaced knees have consistently reported the LCL to be tightest in full extension, and to relax as the knee flexes,^22^,^49^ which is in agreement with the elongation patterns presented in our study. However, compared to the LCL, the biomechanics of the MCL is more complicated mainly due to its band-like geometry. Early investigations on natural cadaveric knees considered the MCL as a homogenous structure and reported the bulk of the MCL to be tense in full extension and progressively more lax with the knee flexion.^24^,^49^ However, using multiple strain sensors, Warren *et al*.^50^ and Arms *et al*.^2^ demonstrated distinctly different strain patterns of the MCL fibers indicating continuous shortening of the posterior fibers and lengthening of the anterior fibers with increasing knee flexion angle. While the majority of previous cadaveric investigations have been performed during passive knee flexion, a few studies have assessed MCL function during loaded knee flexion. Using robotic manipulators, Wijdicks *et al*.^51^ as well as Kanamori *et al*.^23^ found the greatest contribution of the MCL in restraining anterior tibial loads and valgus moments applied to the healthy cadaveric knees at 60° of knee flexion. Similarly, Athwal et al.^4^ tested cadaveric knees implanted with cruciate retaining and posterior stabilized implants, and found the maximum contribution of the superfacial MCL in restraining anterior tibial force as well as internal external rotation moments to be at 50–60° joint flexion. The results from our current study support these previous data on the distinct elongation patterns of the MCL bundles. In particular, we found the anterior region of the MCL as the only portion stretching with increasing the knee flexion; hence highlighting the role of the aMCL in maintaining postoperative knee joint stability in flexed postures. Moreover, the MCL elongation patterns observed in this study indicate the highest aMCL elongation at 50–60° knee flexion, therefore concurring with previous data reported on the MCL restraining function in natural and replaced knees. Clinically, as noted in previous studies,^21^,^46^ these findings suggest that excessive release of the aMCL should be avoided intraoperatively in order to reduce the risk of mid-flexion instability after TKA.

To overcome the invasive nature and technical limitations^10^,^16^ of sensor-based studies, image-based assessment of the ligament elongation patterns has recently been the focus of attention. Here, stationary fluoroscopy together with 3D modelling of the knee joint have been used to estimate the *in vivo* length-change patterns of the collateral ligament bundles during a forward-lunge and the stance phase of the level walking, and reported flexion-dependent patterns both in healthy and TKA subjects.^29^,^37^ Our results revealed similar elongation patterns to these studies, but extend upon the previous literature to confirm that in TKA subjects with an ultra-congruent design, the LCL and pMCL shorten with increasing knee flexion angle, whereas the aMCL elongates until mid-flexion range and becomes shorter thereafter. Importantly, these elongation patterns have now been assessed throughout complete activity cycles, revealing that elongation of all bundles appears to be almost completely independent of activity type, with knee flexion angle clearly being the dominant guiding parameter. This supports results of our previous *in silico* investigation indicating lack of significant task-dependency in the ligament elongation patterns for the knees replaced with three different implant designs.^20^ However, flexion angle does not entirely explain the elongations of the collateral ligaments. We found a slightly greater aMCL elongation during level walking compared to the other activities, despite the peak flexion angle being the lowest (Fig. 3). This can be explained by the distinctive differences between the internal-external rotation patterns of the studied activities. In particular, at peak knee flexion angle during squat, the joint is about 8° externally rotated (compared to the neutral rotation alignment for the level walking).^43^ This excessive external tibial rotation can reduce the maximum aMCL elongation at peak knee flexion during squat as confirmed by our previous sensitivity analysis.^20^ However, it is critical to note that this study has investigated subjects with an ultracongruent TKA implant that is known to constrain the joint kinematics,^45^ producing tibio-femoral motion that is highly flexion dependent. Other knee implant designs have been shown to present more task-dependent kinematic patterns,^44^ plausibly due to the additional geometric freedom allowed by the implant. Moreover, no pre-operative analysis of these subjects was available. As a result, degeneration of their knee ligaments could not be excluded, which might have influenced post-TKA ligament function.^14^ In this respect, further investigations into the dynamic functionality of healthy vs. osteoarthritic knees are clearly required before a more general understanding of physiological elongation patterns of the MCL and LCL can be elucidated.

While image-based investigations of the ligament elongation patterns (including this study) have provided critical insights into the function of the collateral ligaments in living human subjects, a sensitivity study to quantify errors induced into the ligament elongation patterns by the uncertainty in the ligament attachment sites has been clearly lacking. Importantly, a number of parameters including the choice of imaging plane, scan quality and resolution, and the observer’s anatomical knowledge are known to affect the accuracy of the ligament attachment sites obtained from medical images.^7^,^40^,^41^ The results of our sensitivity analysis indicate that collateral ligament elongation patterns are highly sensitive to variations in the femoral attachment sites. In particular, we observed an increase of 0.38% in maximum aMCL length when its femoral attachment was moved by 1 mm in the anteroposterior direction. These data suggest that even with ligament attachments extracted directly from MR images (with an average MCL femoral attachment site identification error of about 4 mm^41^), the elongation patterns may contain errors of up to 1.5% of the ligament reference length that need to be considered when interpreting the results.

Implantation of the TKA components relative to the native anatomy may vary substantially depending on the surgical technique.^11^,^27^ This investigation revealed that MCL and LCL elongation patterns are substantially more sensitive to the location of the femoral attachment sites relative to the femoral component than the tibial attachments relative to the tibial component. Thus, in TKA with an ultra-congruent design where inlay geometry dictates the relative tibiofemoral kinematics, the implantation of the femoral component will critically govern post-operative ligament elongation. These findings are consistent with those reported by Fitzpatrick *et al*.^15^ using a probabilistic finite element approach, and can partially explain the flexion instability reported in patients with malrotated femoral components.^6^,^36^

A number of limitations to this study need to be considered when interpreting the presented results. Our study is based on the measurement of only six patients, each implanted with an ultra-congruent TKA design that is known to limit the kinematics of the knee joint,^45^ thus plausibly affecting the elongation patterns observed in this cohort. As such, these data provide a clearly defined baseline for further benchmarking, but further investigations are clearly required in order to provide a full understanding of collateral ligament function with other implants, and in healthy knees. Moreover, we captured a number of activities with limited ranges of knee flexion and the reported findings might therefore not be generalizable to patients with other implant designs and activities that involve deeper knee flexion angles. One important aspect to consider in any investigation into elongation patterns of passive soft tissue structures is that the zero-strain condition of the ligament fibers remains unknown.^19^ To deal with this important issue, all ligament elongation data were normalized to the length of the ligament fibers at heel strike of the level gait cycles. As a consequence, the reported ligament elongation patterns can only be subjectively compared to those obtained with a different choice of reference length. Finally, the simplified wrapping surfaces used in the OpenSim environment might have slight deviations from the original bone geometry that may consequently introduce minor errors in the ligament length-change analysis.

In conclusion, for the first time, this study has revealed the elongation patterns of the collateral ligament bundles in TKA patients throughout complete functional activities of daily living, while also confirming the previously observed distinct elongation characteristics of the different bundles. Regardless of the activity type, close to isometric behavior of the intermediate bundle, lengthening of the anterior bundle, and shortening of the posterior fibers of the MCL were observed with increasing knee flexion angle. These data emphasize the importance of the anterior fibers of the MCL to support postoperative knee stability and suggest that these fibers are carefully considered during ligament balancing in TKA to prevent them from over-stretching or hyper-laxity during dynamic activities. Moreover, sensitivity of the ligament elongation patterns to the location of their femoral attachment sites relative to the femoral component highlights the critical role of femoral component alignment in governing the ligament elongation patterns after TKA with ultra-congruent implant designs.

## Appendix

**Table A1:** Fmax and F-statistics (in bracket) for the SPM tests performed to assess task-dependency of the ligament elongation patterns

	LW vs. DW	LW vs. SD	LW vs. SQ	DW vs. SD	DW vs. SQ	SD vs. SQ
LCL	5.70 (23.85)	5.65 (21.97)	1.51 (16.30)	21.07 (30.71)	0.93 (26.85)	2.90 (29.42)
aMCL	2.72 (21.19)	1.94 (20.08)	1.88 (13.93)	5.51 (29.46)	15.69 (23.71)	14.39 (27.31)
iMCL	2.36 (21.34)	1.72 (20.14)	1.42 (13.94)	2.84 (29.95)	9.58 (23.86)	7.06 (27.07)
pMCL	2.05 (21.55)	1.49 (20.27)	1.06 (13.95)	1.63 (30.47)	5.87 (23.97)	3.32 (26.79)

LW, DW, SD and SQ represent level walking, downhill walking, stair decent and squat
